# The Drosophila Transcription Factors Tinman and Pannier Activate and Collaborate with Myocyte Enhancer Factor-2 to Promote Heart Cell Fate

**DOI:** 10.1371/journal.pone.0132965

**Published:** 2015-07-30

**Authors:** TyAnna L. Lovato, Cheryl A. Sensibaugh, Kirstie L. Swingle, Melody M. Martinez, Richard M. Cripps

**Affiliations:** Department of Biology, University of New Mexico, Albuquerque, NM 87131–1091, United States of America; New York Medical College, UNITED STATES

## Abstract

Expression of the MADS domain transcription factor Myocyte Enhancer Factor 2 (MEF2) is regulated by numerous and overlapping enhancers which tightly control its transcription in the mesoderm. To understand how *Mef2* expression is controlled in the heart, we identified a late stage *Mef2* cardiac enhancer that is active in all heart cells beginning at stage 14 of embryonic development. This enhancer is regulated by the NK-homeodomain transcription factor Tinman, and the GATA transcription factor Pannier through both direct and indirect interactions with the enhancer. Since Tinman, Pannier and MEF2 are evolutionarily conserved from *Drosophila* to vertebrates, and since their vertebrate homologs can convert mouse fibroblast cells to cardiomyocytes in different activator cocktails, we tested whether over-expression of these three factors in vivo could ectopically activate known cardiac marker genes. We found that mesodermal over-expression of Tinman and Pannier resulted in approximately 20% of embryos with ectopic *Hand* and *Sulphonylurea receptor (Sur)* expression. By adding MEF2 alongside Tinman and Pannier, a dramatic expansion in the expression of *Hand* and *Sur* was observed in almost all embryos analyzed. Two additional cardiac markers were also expanded in their expression. Our results demonstrate the ability to initiate ectopic cardiac fate in vivo by the combination of only three members of the conserved *Drosophila* cardiac transcription network, and provide an opportunity for this genetic model system to be used to dissect the mechanisms of cardiac specification.

## Introduction

Human heart defects are the most common form of congenital birth defects, and a leading contributor to morbidity and mortality in later life [1,2]. A detailed understanding of the molecular events that control the specification and formation of the circulatory system is critical to defining the mechanisms of such diseases, and their potential therapies.

Research over the last 20 years has identified a detailed transcriptional regulatory network that controls the formation of the mammalian heart, where there is extensive auto- and cross- regulation of component transcription factor genes [3]. Importantly, key factors in the regulatory network are now known to be encoded by genes for which mutations cause heart defects in humans. Such network components include the NK homeodomain factor NKX2.5, with mutations leading to malformed cardiac structures, cardiomyopathy and irregular conduction [4–9]; the GATA factor GATA4, with mutations leading to septal defects [10]; and the MADS domain transcription factor Myocyte enhancer factor-2 (MEF2), with mutations leading to inherited coronary artery disease [11]. Clearly, defining transcriptional networks for complex biological processes provides important insight into the mechanisms of diseases affecting that process.

In the cardiac tissue, a knowledge of the regulatory network controlling cardiac formation can also be exploited for therapeutic purposes. One potent avenue for treatment of heart disease is to supply therapeutic cells to repair the damaged tissue [12–16]. Source cells for such treatment could include fibroblasts from the host that have been induced to a cardiac fate by expression of cardiac specification transcription factors. Indeed, several recent studies have identified cocktails of regulatory factors that can promote cardiac fate in fibroblasts. Moreover, many of the successful studies include expression of the central cardiac regulatory factors NKX2.5, GATA4 and MEF2 [17–21].

The cardiac regulatory network is also strongly conserved across evolution, with many of the component genes and regulatory pathways present in Drosophila [3]. This makes the simpler Drosophila system, that has less genetic redundancy amongst cardiac regulatory factors, an important model for defining basic regulatory interactions in cardiac development. In *Drosophila*, the heart is comprised of two distinct cells types that can be characterized by their mutually exclusive expression of the NK-homeodomain transcription factor Tinman or the orphan steroid hormone receptor Seven-up. Whereas Tin and Svp cell types perform distinct function in the mature organ, the cells are still contractile, and express similar groups of contractile protein isoforms [22–24]. Accordingly, both cardiac cell types express *Mef2*, which is required for muscle protein gene expression in all contractile heart cells [25–26].

Previous studies have identified direct regulatory interactions, at least in a subset of cardial cells, between the NKx2.5 ortholog Tinman (Tin), the GATA4 ortholog Pannier (Pnr), and Drosophila MEF2 [27–30]. In particular, three enhancers have been identified that regulate *Mef2* transcription in the heart. The first enhancer is active early, by stage 11 of embryogenesis, and controls expression in Tinman-expressing dorsal mesodermal cells and cardiac precursors [27, 30]. This enhancer is activated by Tinman and the zinc finger transcription factor Pannier, which maintain enhancer activity through the end of embryogenesis. A second enhancer controls *Mef2* expression in Seven-up expressing cells [31], and a third enhancer becomes active in the somatic mesoderm and both cardiac cell types at stage 14 of embryogenesis [32]. A portion of the third enhancer responsible for somatic mesodermal expression was found to be regulated by a member of the Gli superfamily of transcription factors, named Lame duck [33–34]. While this latter enhancer was also recently shown to bind Tinman in chromatin immunoprecipitation assays [35], sequences critical to its regulation have yet to be investigated.

Here, we have identified the transcription factors that regulate the cardiac expression of this third *Mef2* cardiac enhancer. The regulation of this enhancer is unique in that it is active in Tin plus Svp cell types of the heart, whereas the previous enhancers were active either in the Tinman expressing cardiac cells or the Seven-up expressing cardiac cells. Our data demonstrate that the enhancer is activated directly by Tinman and Pannier. These findings support the idea that proper heart development requires the fine-tuning of MEF2 protein expression before hatching to the larval stage. In addition, we also demonstrate that Tin and Pnr can work with MEF2 protein to activate downstream genes in the cardiac program, similar to the roles of their mammalian counterparts in conversion of fibroblasts to a cardiac fate. Our in vivo studies underline the potent cardiogenic activities of these factors, and uncover the potential of Drosophila to define mechanisms by which naïve cells can be converted into cardiomyocytes.

## Materials and Methods

### Generation of promoter-*lacZ* constructs

The 345bp enhancer was PCR-amplified using the forward primer (5’-CCTCTCTTTTGGCAGAAAGTCG-3’) and reverse primer (5’-AAACTCATCTCCACGCCACTGC-3’). The product was cloned into the vector pLacZattb and injected into flies using phiC31 integrase at the landing site 86Fb [36]. Mutation of the Tinman and Lame duck consensus sequences was carried out by PCR site directed mutagenesis [37]. Primers for each of the constructs were designed to contain an *EcoRI* site in place of the consensus binding sequences. Primary PCR amplification products using the original forward primer/reverse mutated primer and the original reverse primer/mutated forward primer were used as templates in a secondary PCR amplification of the full-length enhancer. The forward Tinman mutant primer was (5’GAGTCGAAATGAATTCGCTGAACTGAACTTC3’) and the reverse Tinman mutant primer was (5’GAAGTCAGTTCAGCGAATTCATTTCGACTC3’). The forward Lame duck mutant primer was (5’TTTGAATGAGATTTATGAAAGAATTCAAAACATCATC’3) and the reverse Lame duck mutant primer was (5’- GATGATGTTTTGAATTCTTTCATAAATCTCATTCAAA-3’). The introduced *EcoRI* sites are in italics. Generation of transgenic flies carrying the mutated enhancers was carried out as previously described [38].

### Immunohistochemistry

Embryos were collected and fixed according to [39]. We used the antibodies described in Table 1.

**Table 1:** Antibodies used in this study, their sources and dilution.

Primary Antibody	Dilution	Source	Secondary Antibody	Dilution	Source
Mouse anti- β-galactosidase	1:400	Promega (Madison, WI)	Vector anti-mouse (Vectastain Elite Kit)	1:500	Vector Laboratories (Burlingame, CA)
Mouse anti-β-galactosidase	1:400	Promega (Madison, WI)	Alexa 488 anti-mouse	1:2000	Molecular Probes (Eugene, OR)
Rabbit anti-MEF2	1:1000	Lilly et al., 1995	Alexa 568 anti-rabbit	1:2000	Molecular Probes (Eugene, OR)
Mouse anti-Fasciclin III	1:500	Developmental Studies Hybridoma Bank (University of Iowa)	Alexa 488 anti-mouse	1:2000	Molecular Probes (Eugene, OR)
Mouse anti-Pericardin	1:50	Developmental Studies Hybridoma Bank (University of Iowa)	Alexa 568 anti-mouse	1:2000	Molecular Probes (Eugene, OR)
Guinea pig anti-H15	1:2000	[40]	Alexa 488 anti-guinea pig	1:2000	Molecular Probes (Eugene, OR)

### In situ hybridization

Embryos were prepared according to [41] until the hybridization step, after which the Watakebe et al. (2010) protocol was followed for labeling and hybridization. PCR primers were used to amplify, from *y w* embryo cDNA, portions of each transcript to be analyzed. PCR products were cloned into the pGEM-T Easy vector (Promega). Insert orientation was determined by sequencing. Plasmids were then linearized at the 5’ end of the transcript and the appropriate RNA polymerase (either T7 or SP6) was used to generate an anti-sense FITC RNA probe to each transcript according the protocol in [42]. Primers used are described in Table 2.

**Table 2:** Sequences of oligonucleotide primers used to amplify cDNA for the indicated genes.

Transcript	Forward primer 5’-3’	Reverse primer 5’-3’
*Hand*	ATGTTTAAGAATTCCGTTGCC	CGTGCGGCCCTTGGTCG
*Sur*	CCGCCATTTCGTGTGTTTGT	GTGGTTGCCTCATAGTGCCT

### Electrophoretic mobility shift assay

Complementary DNA oligonucleotides were ordered from Sigma-Aldrich (St. Louis, MO) to generate double stranded DNA molecules with 5’ GG overhangs. The oligonucleotides were radioactively labeled with 32P-dCTP (Perkin Elmer, Waltham, MA) using Klenow enzyme (New England Biolabs, Beverly, MA). The sequence tested was Tin1 5’-GG-GAGTCGAAATCACTTGAGCTGAACTGA-3’. Mutant Tin competitor oligonucleotides were the same as wild type, but with the *EcoRI* site replacing the Tin site, and used for mutagenesis. Tinman protein was synthesized in vitro from pBSK-Tin [43] using T3 polymerase in the Promega TNT Coupled Reticulocyte Lysate System (Promega, Madison, WI),

### Cell culture co-transfection assay

Tinman and Pannier cDNAs were cloned into the pPacPl plasmid, and the late stage enhancer was cloned into CHAB [44]. Drosophila Schneider’s line 2 cells (SL2 or S2; Drosophila Genomics Resource Center) cells were grown in Schneider’s medium supplemented with 10% fetal bovine serum (Invitrogen Corp., Carlsbad, CA) at 25°C. Transfections were carried out with TransIT Transfection Reagent (Mirus, Madison, WI) according to the manufacturer’s directions. Experiments were carried out in triplicate and the average activation fold, standard error and t-tests were calculated to assess significance.

### Fly stocks and crosses

The *69B-gal4* line and *UAS-pnr* lines were obtained from the Bloomington Stock Center. The *UAS-tin* line was described previously [45]. The *UAS-tin* and *UAS-pnr* transgenes are both on chromosome 2, therefore the *UAS-tin UAS-pnr* line was generated by recombination. We incorporated our enhancer line, which is located on the third chromosome, into this stock using standard genetic techniques.

## Results

### *Mef2* contains a cardiac enhancer expressed in all cell types of the cardiac tube

**Fig 1:**
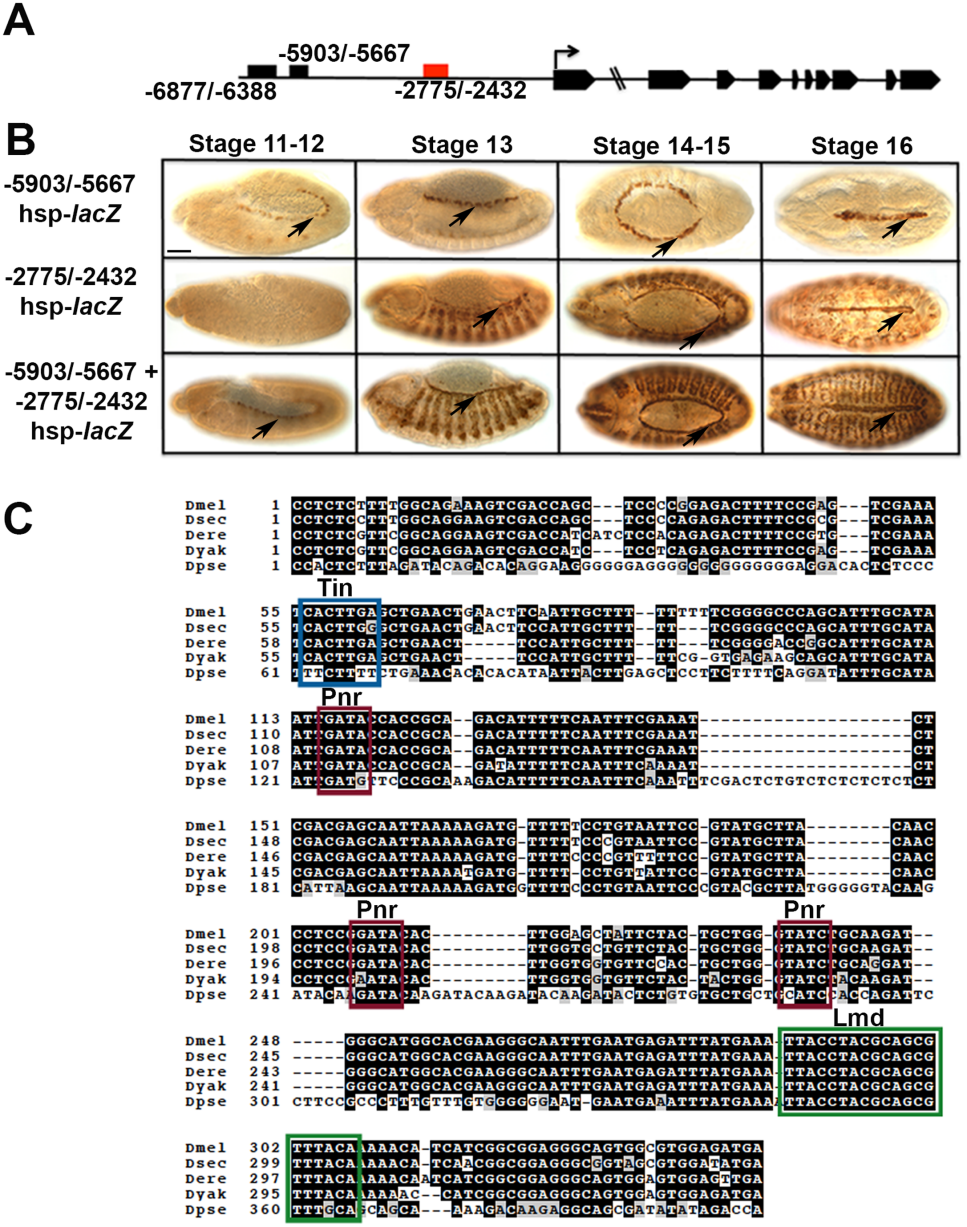
Identification of a proximal *Mef2* Cardiac Enhancer. (A)Diagram of the *Mef2* gene and its cardiac enhancers. The most distal enhancer (-6877/-6388) refers to the Seven-up cell enhancer. -5903/-5667 represents the Tinman-dependent enhancer and the red box (-2775/-2432) is the late stage cardiac and somatic mesodermal enhancer characterized here. (B) Activity of *Mef2* cardiac enhancers fused to *lacZ* reporters. The embryos were stained for β-Gal accumulation. Top row, the -5903/-5667 enhancer was active earliest in development, and reporter activity was detected until stage 16. Middle row, the -2775/-2432 enhancer became active at stage 13 in cardiac cells (arrow) and skeletal myoblasts, and was active in all cardiac cells by stage 16. Bottom row, when the two enhancers were fused, there was reporter expression in all cardiac cells during embryogenesis. Arrows indicate heart cells. Bar, 100μm. (C)Alignment of the proximal enhancer sequence with four *Drosophila* species. A conserved Tinman binding site is marked by a blue box, Pannier sites are marked with red boxes, and the proposed Lame duck binding site identified by Duan et al [33] is marked with a green box.

We have worked to isolate the cardiac specific portion of the large late stage enhancer first identified by Nguyen and Xu [32], which lies more proximal to the transcription start site of the *Mef2* gene than the previously identified cardiac enhancers (Fig 1A and S1 Fig). We generated PCR fragments from this region, cloned them into a plasmid containing a *lacZ* reporter gene and generated transgenic flies that contained the *Mef2-lacZ* constructs. Embryos from these lines were stained with an antibody against β-Galactosidase to visualize activity of the enhancer. The smallest enhancer fragment with complete activity in both cardiac cell types lies at -2432/-2775 relative to the *Mef2* transcription start site. This 345-bp fragment also contained the 170bp mesodermal enhancer characterized by Duan et al [33] and a portion of the mesodermal enhancer characterized by Busser et al ([34] that had additional regulatory regions lying downstream of our enhancer. Attempts to separate the cardiac and somatic enhancer activities by 5’ or 3’ deletions resulted in loss of activity from both the cardiac cells and somatic mesoderm (data not shown). This observation indicated that certain enhancer sequences are utilized in both cardiac and skeletal muscle tissues.

The previously characterized -5903/-5667 Tin-responsive enhancer becomes active early in development when the cardiac cells are becoming specified at stage 11 [27,30]. Its activity becomes restricted to the Tin expressing cells by stage 14 and remains active until the end of embryogenesis (Fig 1B, row 1). By contrast, the enhancer characterized here becomes active in cardiac cells at stage 14 and remains active until the end of embryogenesis (Fig 1B, row 2). Interestingly, when we fused the -2432/-2775 late stage enhancer to the early Tin specific enhancer, we observed that the combined enhancer completely recapitulated MEF2 expression in the heart from the earliest stages to the end of embryogenesis in all cardiac cell types (Fig 1B, row 3).

To gain insight into how expression of *Mef2* is regulated via the late enhancer, we analyzed the sequence for known transcription factor binding sites, and observed consensus sequences for Tin and Pnr (Fig 1C). These two factors have already been shown to activate *Mef2* in the heart via the -5903/-5667 enhancer, and their consensus binding sites have also been shown to cluster together within numerous cardiac gene promoters [35]. We therefore tested the ability of Tin and Pnr to activate the enhancer in vitro.

### Tinman and Pannier activate the enhancer in vitro, and Tinman is capable of binding to its consensus sequence within the enhancer

**Fig 2:**
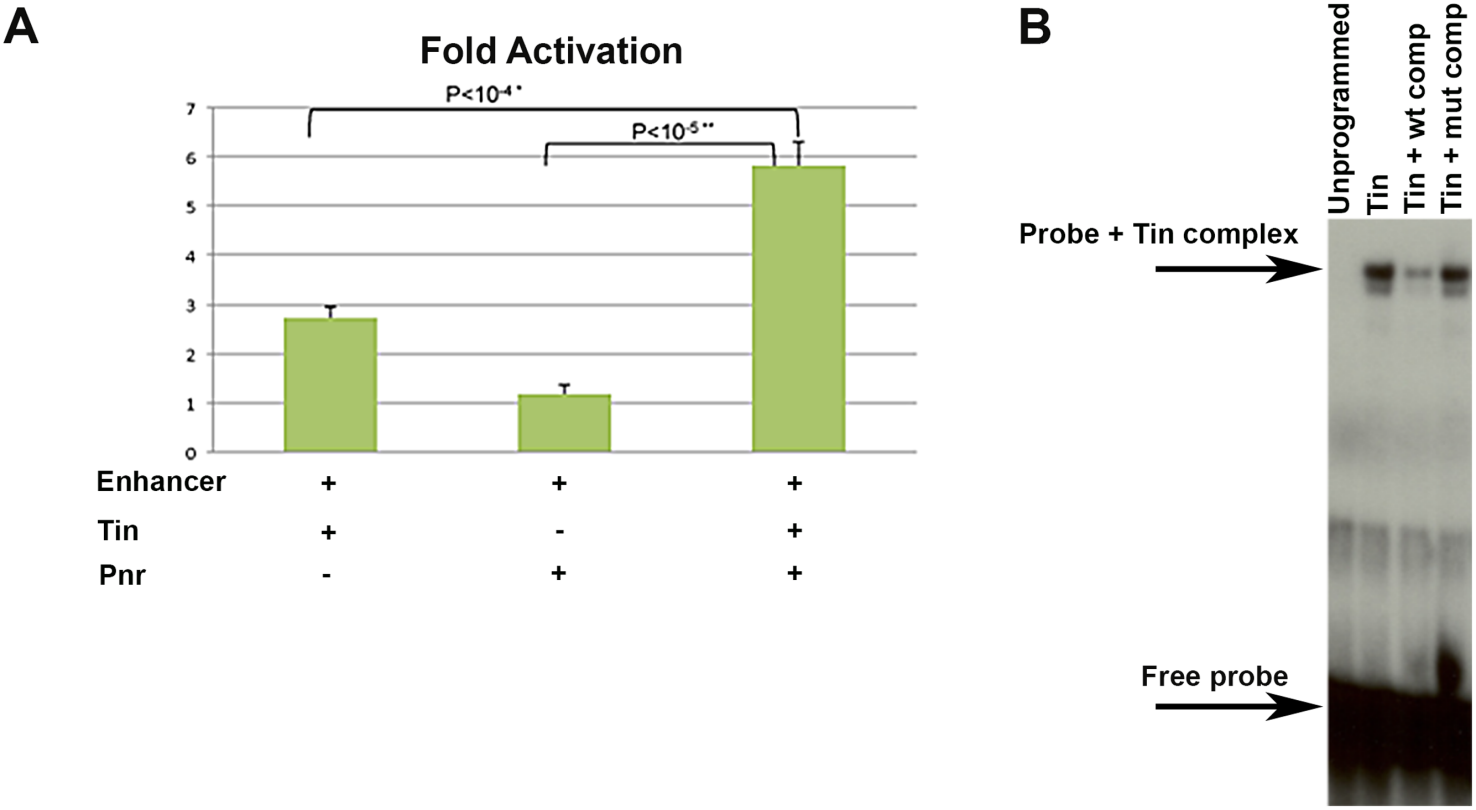
Tinman and Pannier activate the enhancer in vitro and Tinman is capable of binding to its consensus sequence within the enhancer. (A)Activation of the -2775/-2432 *Mef2-lacZ* in S2 cells by Tin, Pnr or Tin combined with Pnr. Tin activated the reporter moderately, while activation by Pnr was not significantly above negative controls. When combined, activation of the reporter was increased significantly above that achieved by Tin alone. (B)Electrophoretic mobility shift assay to determine if Tin and Pnr could bind to consensus sites. Free probe had a high mobility when combined with unprogrammed lysate (Un). A complex of probe plus protein was formed in the presence of Tin lysate, which was competed by 300X excess of nonradioactive wild-type sequence (wt comp) but not by 300X excess of nonradioactive mutant sequence (mut comp).

To test the ability of candidate factors to regulate the *Mef2* enhancer, we transfected *Drosophila* S2 cells with the enhancer fused to a *lacZ* reporter gene, along with plasmids containing the cDNAs of either *tin*, or *pnr*, or both factors. After incubation for 48hr, cell lysates were prepared and reporter activity was determined using a quantitative ßGal assay. There was moderate but significant activation of the *Mef2-lacZ* construct by Tin, while Pnr on its own was unable to significantly activate the enhancer. When Tin and Pnr were combined, activation was more than additive, suggesting that the two factors might work synergistically to activate *Mef2* in the heart (Fig 2A).

Next, we tested the ability of Tin to bind to the *Mef2* enhancer in an electrophoretic mobility shift assay, using in vitro translated protein and radioactively labeled DNA. Tin bound to the consensus site within the enhancer robustly, as visualized by the presence of a protein plus probe complex in the assay (Fig 2B). The interaction with Tin was specific, because unlabeled wild-type competitor was able to compete away binding, while unlabeled mutant competitor (that had the consensus site replaced with an *EcoRI* site) was unable to reduce binding. Pnr was unable to activate the enhancer on its own in cell culture and we were also unable to detect an interaction in our binding assays (data not shown), which initially suggested to us that Pnr might not bind to the enhancer directly.

### The Tinman and Pannier binding sites are required for complete enhancer activity in vivo

**Fig 3:**
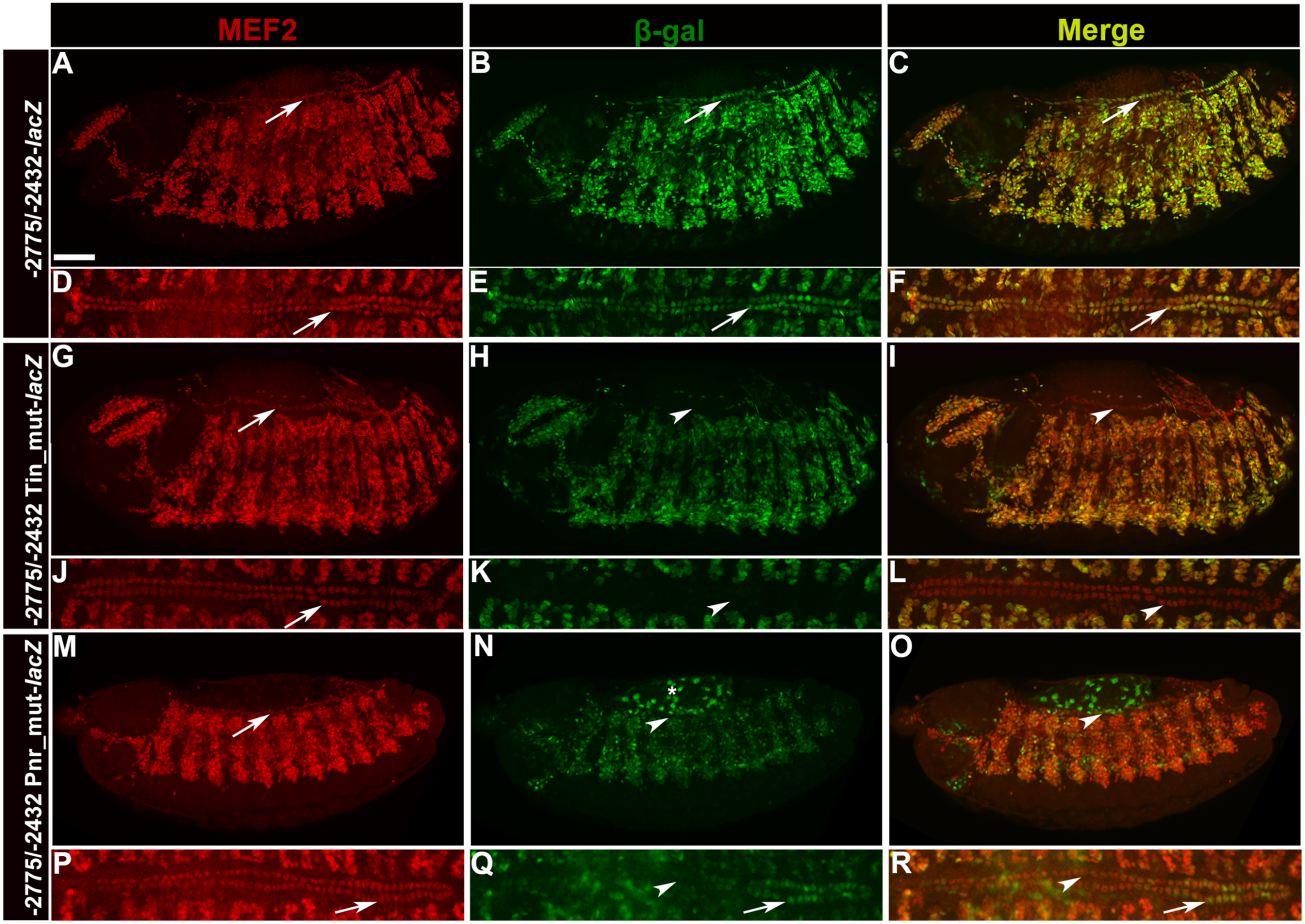
Mutation of the Tinman consensus site in the -2775/-2432 enhancer results in loss of enhancer activity in cardiac cells. (A-F)-2775/-2432 *Mef2-lacZ* embryos at stages 14 (A-C) and 16 (D-F). (A,D)Antibody stain against MEF2. MEF2 could be detected in all cells of the heart and throughout the somatic mesoderm. (B,E)Antibody stain against β-Galactosidase. Activity of the late stage enhancer was almost identical to that of *Mef2* expression. In (B), the enhancer is just becoming active in the heart cells, therefore a few cells lacked activity at this stage. By stage 16 (E) all cardiac cells showed ßGal accumulation. (C)Merge of (A) and (B); (F)Merge of (D) and (E). (G-L)Embryos carrying the -2775/-2432 *Mef2-lacZ* enhancer with the Tinman consensus site mutated, at stages 14 (G-I) or 16 (J-L). (G,J)Antibody stain against MEF2. MEF2 marks all cells of the heart and the somatic mesoderm. (H,K)Antibody stain against β-Galactosidase. Activity of the mutated enhancer was completely lost from the cardiac cells, and was slightly reduced in the somatic mesoderm. (I)Merge of (G) and (H). (L)Merge of (J) and (K). Arrows point to MEF2 positive cardiac cells, arrowheads point to the same cells lacking β-Galactosidase. (M-R) Embryos carrying the -2775/-2432 *Mef2-lacZ* enhancer with the three Pannier consensus sites mutated, at stages 14 (M-O) or 16 (P-R). (O)Merge of (M) and (N). (R) Merge of (P) and (Q). Arrows point to cardiac cells, arrow heads point to cells that have lost enhancer activity. Asterisk denotes activity in the amnioserosa. Bar, 100μm.

Having determined that Tin could bind to the consensus site in the enhancer, we next determined if the site was required for enhancer activity. Using site-directed mutagenesis, we mutated the Tin consensus site within the context of the full-length enhancer, fused the mutated enhancer to a *lacZ* reporter, and generated transgenic flies carrying this construct. We analyzed reporter expression in transgenic embryos of the wild-type construct (Fig 3A–3F) relative to the Tin-mutant construct. We noted that *lacZ* reporter activity of the mutant construct was slightly reduced in the somatic mesodermal cells, but still present. However, reporter expression was lost from all cells of the heart (Fig 3G–3L). This loss of enhancer activity was apparent at early and late stages of cardiogenesis, suggesting that Tin is a direct and essential activator of this enhancer in vivo during the embryonic stage. In addition, we mutated the three Pnr consensus sites and assessed enhancer-*lacZ* activity in vivo. Here, we saw a reduction of reporter activity in the heart cells (Fig 3M–3R), which suggested that Pnr does have direct interaction with its consensus sites and contributes to enhancer robustness as seen in our cell culture experiments. We were unable to observe a direct interaction between Pnr protein and the enhancer in vitro; however, the mutation of the consensus binding sites in vivo clearly diminished enhancer activity and the synergistic activation of the enhancer with Tinman was robust, suggesting the Pnr does in fact interact directly. In the Pnr-mutant enhancer-*lacZ* lines, we also saw an expansion of enhancer activity in the amnioserosa. We hypothesize that while the Pnr consensus sites are required for full activation of the enhancer in cardiac cells, the sites are also required for repression of *Mef2* in the amnioserosa, as Pnr has been shown previously to act as a repressor of transcription [46].

### Ectopic expression of Tinman and Pannier results in expansion of enhancer activity

To further test the hypothesis that Tin and Pnr are direct and positive activators of *Mef2* expression via this enhancer, we determined if ectopic expression of Tin could expand the activity of our enhancer in embryos. To achieve this, we initially generated embryos carrying the ectodermal, amnioserosal [47] and nervous system Gal4 driver *69B-Gal4*, plus *UAS-tin* and the *Mef2* enhancer-*lacZ*. This combination directed expression of Tin in the ectoderm and ventral nerve cord. If Tin was a direct activator of the enhancer, we predicted that these embryos should show *lacZ* expression in the ectoderm and/or nerve cord. Since the mesodermal expression of the enhancer is quite robust, we directed our attention to the ventral nerve cord to look for expansion of expression of the enhancer, but failed to see any reporter activity in this tissue. We hypothesized that there might be expansion of the enhancer in other areas of the ectoderm that might be difficult to discern, given the intensity of the underlying mesodermal activity of the enhancer.

To reduce the skeletal muscle-specific enhancer activity, we mutated a site previously shown to be required for high levels of somatic mesoderm enhancer activity, and thought to interact with the zinc finger transcription factor Lame duck (Lmd, indicated in Green in Fig 1)[33]. Whether it is Lmd that interacts with this sequence has recently been called into question, since Busser et al [34] identified three Lmd binding sites in this region of the genome, none of which corresponded to the site of Duan et al [33]. Instead, they proposed that a Forkhead domain factor most likely interacts with the sequence that we mutated. In either scenario, our goal was to reduce the overall somatic muscle activity of the enhancer, in order that we could more effectively assess the abilities of Tin and/or Pnr to activate the enhancer.

**Fig 4:**
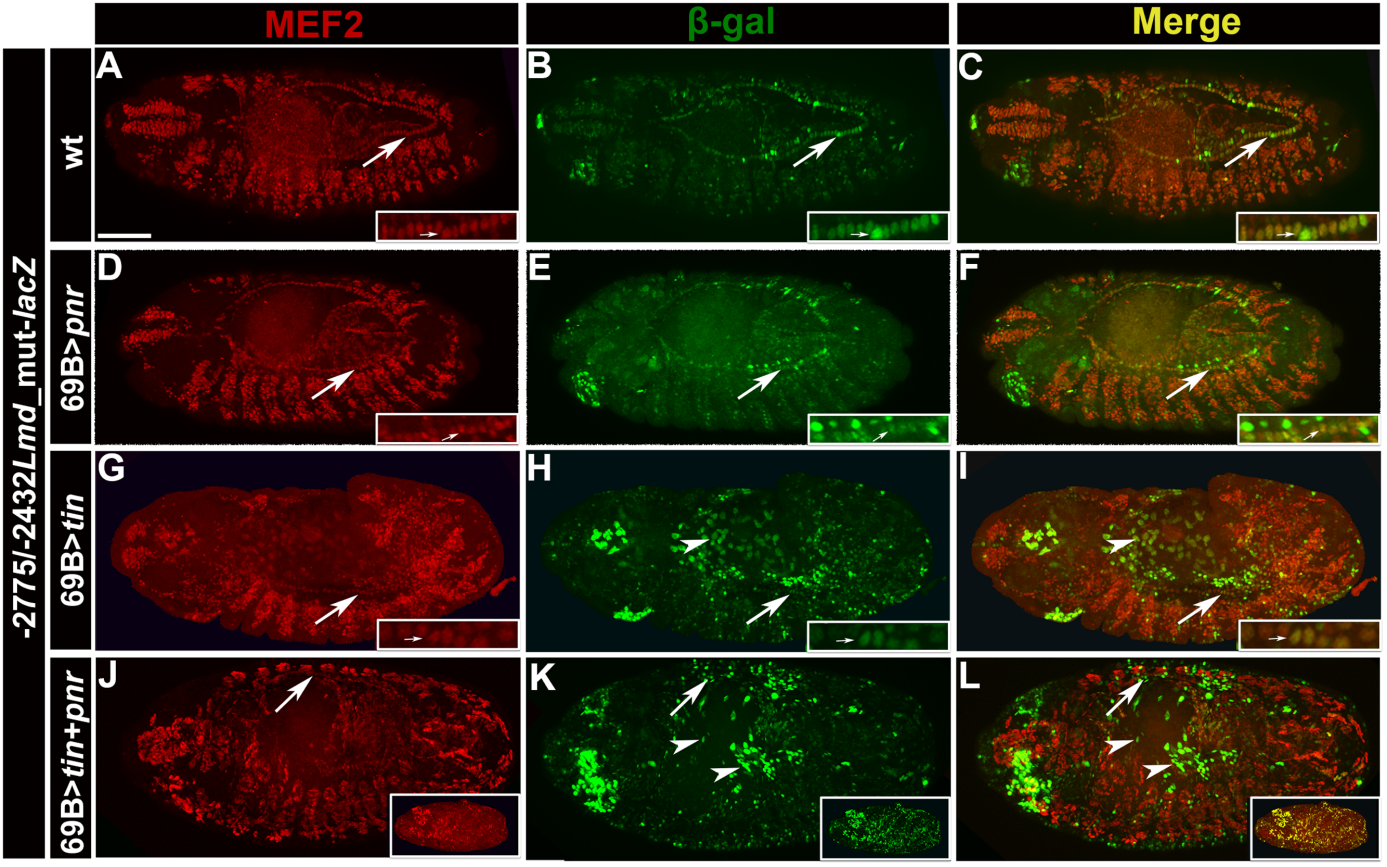
Ectopic expression of *tinman* and *pannier* results in expansion of enhancer activity. All embryos are stage 13–14 embryos carrying the -2775/-2432 *Mef2-lacZ* with the Lame duck consensus site mutated. (Left column) accumulation of MEF2; (center column) accumulation of ß-Gal; (right column) merge of prior two channels. Embryos have ectopic ectodermal expression of the following genes: (A-C)no additional genes expressed, note that activity of the enhancer can be seen in all cells of the heart (arrows), but the somatic mesodermal stain is reduced; (D-F)ectopic *pnr* expression, stains are similar to (A-C); (G-I) ectopic *tin* expression, note that activity of the enhancer is expanded in the ectoderm and amnioserosa (arrowheads). Arrow indicates cardiac cells. (J-L) ectopic *tin* and *pnr* expression, note that the enhancer is more robustly activated in the ectoderm and amnioserosa when compared to (H), which can been seen more dramatically in the inset, and ß-Gal accumulation co-localizes with the MEF2 expression seen in (J). Arrow indicates to cardiac cells. Scale bar, 100μm.

We generated transgenic flies carrying this mutated construct, and found that *LacZ* expression was reduced in the somatic mesoderm, but not completely lost. This result was consistent with the identification by Busser et al [34] of the Lmd binding sites, since the most promoter-distal Lmd site is retained in the -2432/-2775 enhancer, and could contribute to enhancer activity in the somatic mesoderm. However, the activity of the mutated enhancer was sufficiently reduced to enable clear visualization of cardiac enhancer-*lacZ* expression. Interestingly, at stage 16, the cardiac cell activity of the enhancer became inconsistent, with random groups of cells losing expression (Fig 4A–4C compared to Fig 3B). This patchy enhancer activity was consistent with an experiment where we tried to remove the 3’ 170 bp of sequence from the enhancer, that included the site mutated here, and we lost all enhancer activity (data not shown), suggesting that 3’ sequences are critical to enhancer activity.

When we ectopically expressed *pnr* in the ectoderm of these transgenic embryos, there was relatively little impact upon the pattern of *lacZ* reporter expression (Fig 4D–4F). We next repeated the ectopic Tin expression experiment with this new transgenic line, and saw reproducible ectopic expression of *lacZ* in the ectoderm and amnioserosa, demonstrating that Tin could activate the enhancer-*lacZ* in vivo outside of the mesoderm (Fig 4G–4I). Since Pnr and Tin collaborated to activate the *Mef2-lacZ* in tissue culture cells, we also tested whether Tin required Pannier for more robust ectopic activation of the enhancer. We generated embryos carrying *UAS-tin*, *UAS-pnr*, *69B-Gal4*, and the mutated enhancer-*lacZ* line. Ectopic enhancer activity was again observed in the ectoderm and amnioserosa at more robust levels than in the cardioblasts (Fig 4J–4L). We suspect this is due to higher levels of induced *tin* and *pnr* expression in the ectoderm compared to the lower endogenous levels in the mesoderm. We confirmed that this expanded activity was ectodermal, by co-staining with the ectodermal marker Fasciclin III and imaging a single confocal Z-slice to confirm co-localization (S2 Fig). In addition, we observed ectodermal expression of the endogenous *Mef2* gene in many of these embryos (Fig 4J inset), as well as robust expansion of enhancer activity (Fig 4K and 4K inset). These data further supported the hypothesis that Tin and Pnr collaborate in activating *Mef2* via this enhancer.

### Tinman and Pannier activate *Hand and Sur* transcription

**Fig 5:**
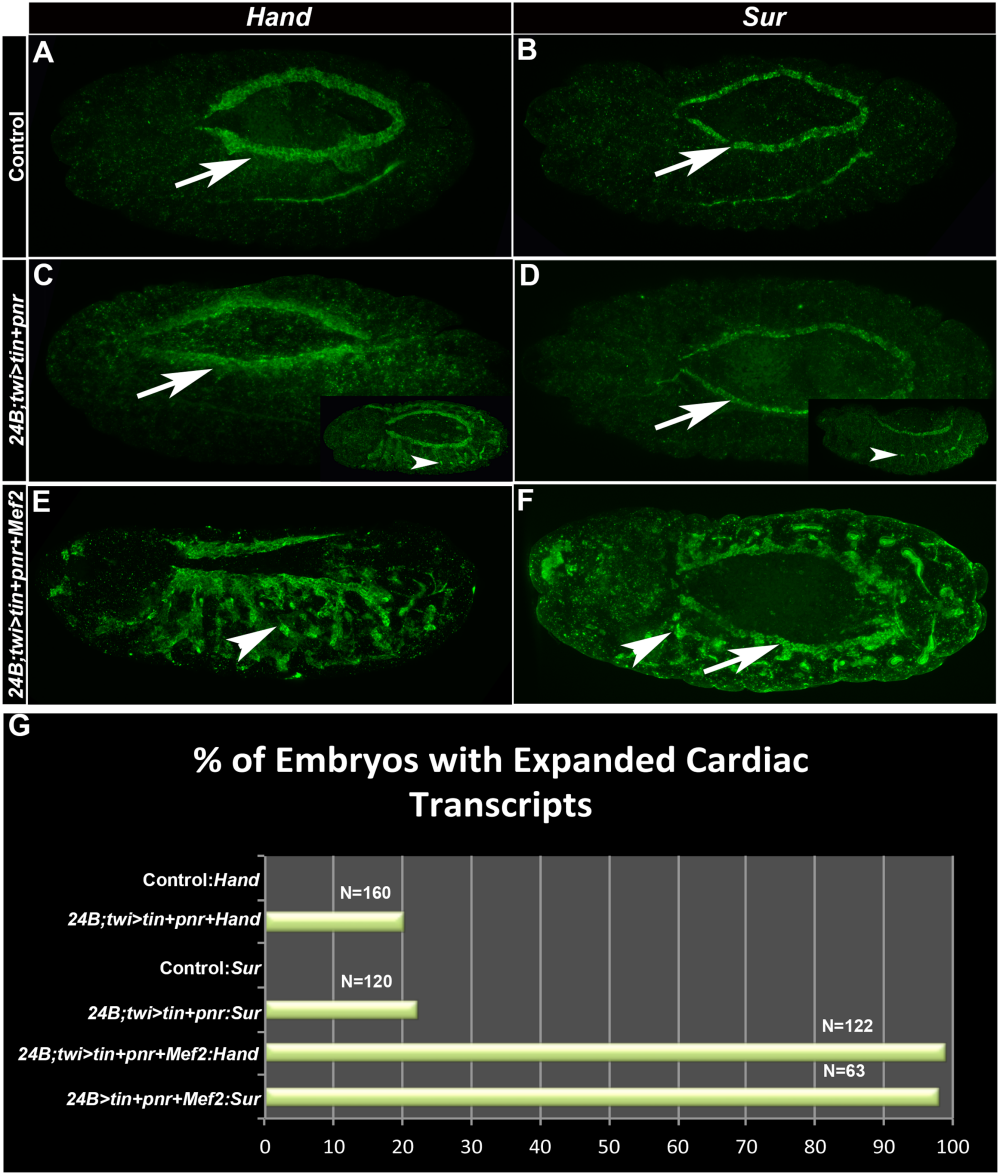
Over-expression of *tinman*, *pannier* and *Mef2* in the mesoderm results in expansion of the expression of cardiac factors. (A-F) Stage 14 embryos of the indicated genotypes stained for *Hand* expression (left column) or *Sur* expression (right column). (A,B)Wild-type expression of *Hand* and *Sur*. (C,D)*24B+twi>tin*+*pnr* embryos. Note that most embryos show normal expression of the cardiac markers, but that a small subset (insets) show expanded expression. (E,F)24B+twi>*tin+pnr+Mef2* embryos. Ectopic *Hand* and *Sur* transcripts were observed in nearly 100% of embryos. Arrows point to wild type expression in the heart, and arrowheads point to expanded expression of *Hand* and *Sur* transcripts in the somatic mesoderm. Bar, 100μm. G: Quantification of effects of over-expression of cardiac transcription factors. Note that expression of *tin* plus *pnr* results in ~20% of embryos showing ectopic marker gene expression, and addition of *Mef2* expression results in almost 100% of embryos showing ectopic cardiac marker expression.

Tin and Pnr working together to activate our enhancer was consistent with several accounts in the literature of their collaboration in activation of *Drosophila* genes [29,48–50] and collaboration of their vertebrate homologs Nkx2.5 and GATA4 [51–54]. Additionally, recent studies in vertebrates have demonstrated that the Tin, Pnr and MEF2 homologs collaborate, alongside other factors, to convert mouse fibroblast cells to cardiac cells [17,19]. We wondered if we could use the *Drosophila* in vivo system in order to investigate this phenomenon further. First, we tested our system by analyzing the effects of only Tin and Pnr over-expression. It had been documented that these factors activate the enhancers of *Hand* and *Sur* [48–49,55] therefore we over-expressed *tin* and *pnr* throughout the mesoderm, and assessed whether they were able to activate transcription of *Hand* and *Sur* compared to the expression of these genes in control embryos (Fig 5A and 5B). In approximately 20% of the embryos, we observed ectopic accumulation of *Hand* or *Sur* transcripts (Fig 5C and 5D). These studies demonstrated that cardiac marker gene expression could be modestly expanded upon over-expression of *tin* plus *pnr*.

### Tinman, Pannier and MEF2 work in collaboration to activate the cardiac program

**Fig 6:**
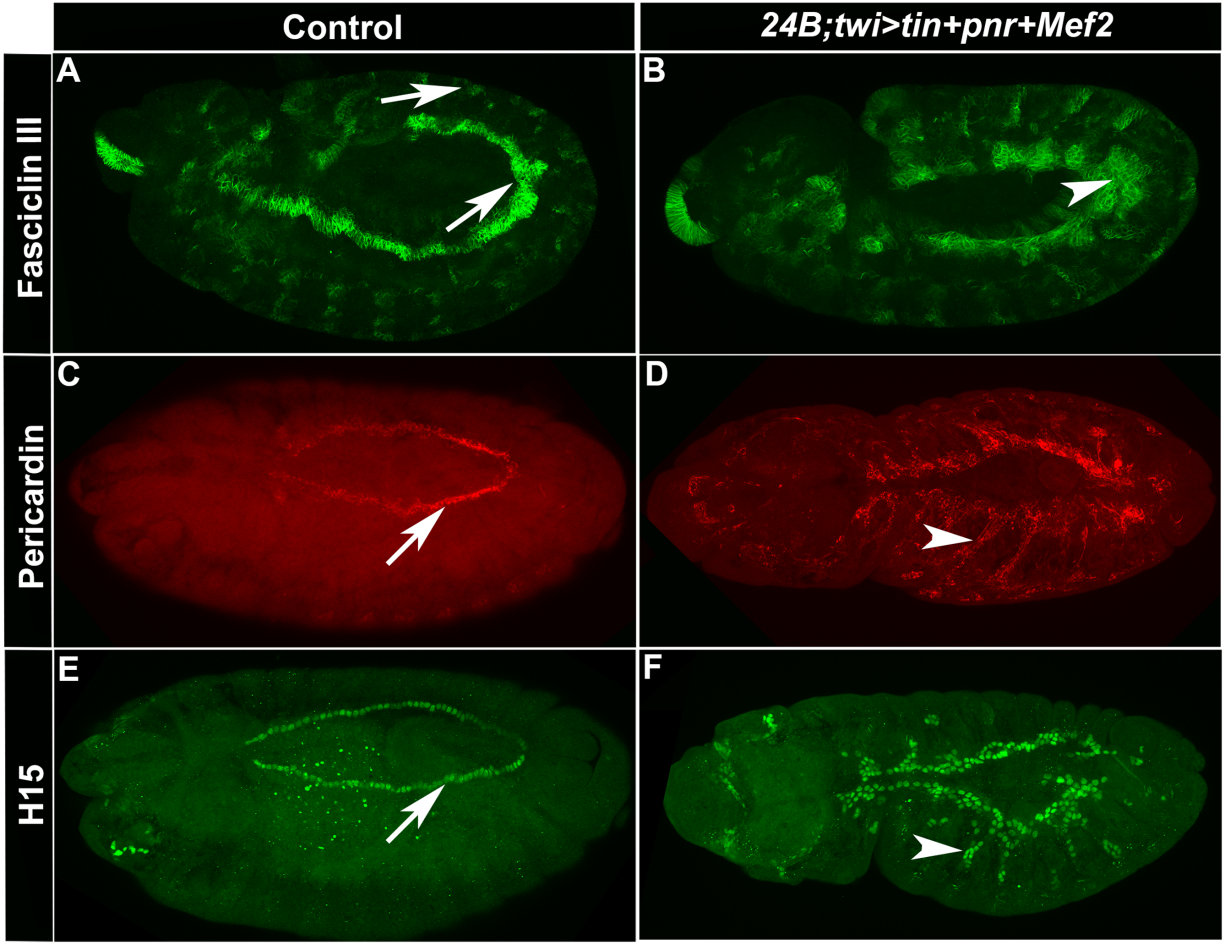
*Pericardin* and *H15* are activated by the over-expression of *tinman*, *pannier* and *Mef2* in the mesoderm. (A,C and E) Control embryos. (B,D and F): 24B+twi>*tin+pnr+Mef2* embryos. (A,B) Antibody stain against Fasciclin III which marks the visceral mesoderm at stage 10. (C,D) Antibody stain against Pericardin which marks the pericardial cells. (E,F) Antibody stain against H15 which is a cardiac-specific T box transcription factor. Arrows point to normal expression and arrowheads point to expanded expression. Bar, 100μm.

We next investigated the effect upon marker gene expression of adding a third factor used in vertebrate conversion experiments, namely MEF2. With the addition of MEF2 to embryos over-expressing Tin and Pnr, the patterns of *Hand* and *Sur* transcripts were dramatically expanded in 100% of embryos analyzed, in a thickening expanse of cells adjacent to where the cardiac tube lies, as well as elsewhere in the mesoderm (Fig 5E and 5F). We also wanted to determine if visceral mesoderm fate was also being expanded in these embryos, since Tin is required for visceral mesoderm specification [43]. To do this, we used an antibody against Fasciclin III, which is expressed in the visceral mesoderm precursors at early embryonic stages. At stage 10, there was a slight expansion of FasIII expression compared to controls (Fig 6A and 6B), however this expansion did not persist into later stages (data not shown). We conclude that the predominant activation of cell fate in these embryos is the activation of cardiac fate.

To further determine if Tin, Pnr and MEF2 were specifically activating the cardiac program, we studied the pericardial cell marker Pericardin (Prc) and an additional cardiac specific marker, H15. Here, we found that Prc and H15 also had expanded expression. This expansion of Prc and H15 was similar to, but not as broad as, the expansion of *Hand* and *Sur* transcripts (Fig 6C–6F). We interpret these results to indicate that MEF2 can potentiate the cardiogenic effects of *tin* and *pnr* in the mesoderm.

**Fig 7:**
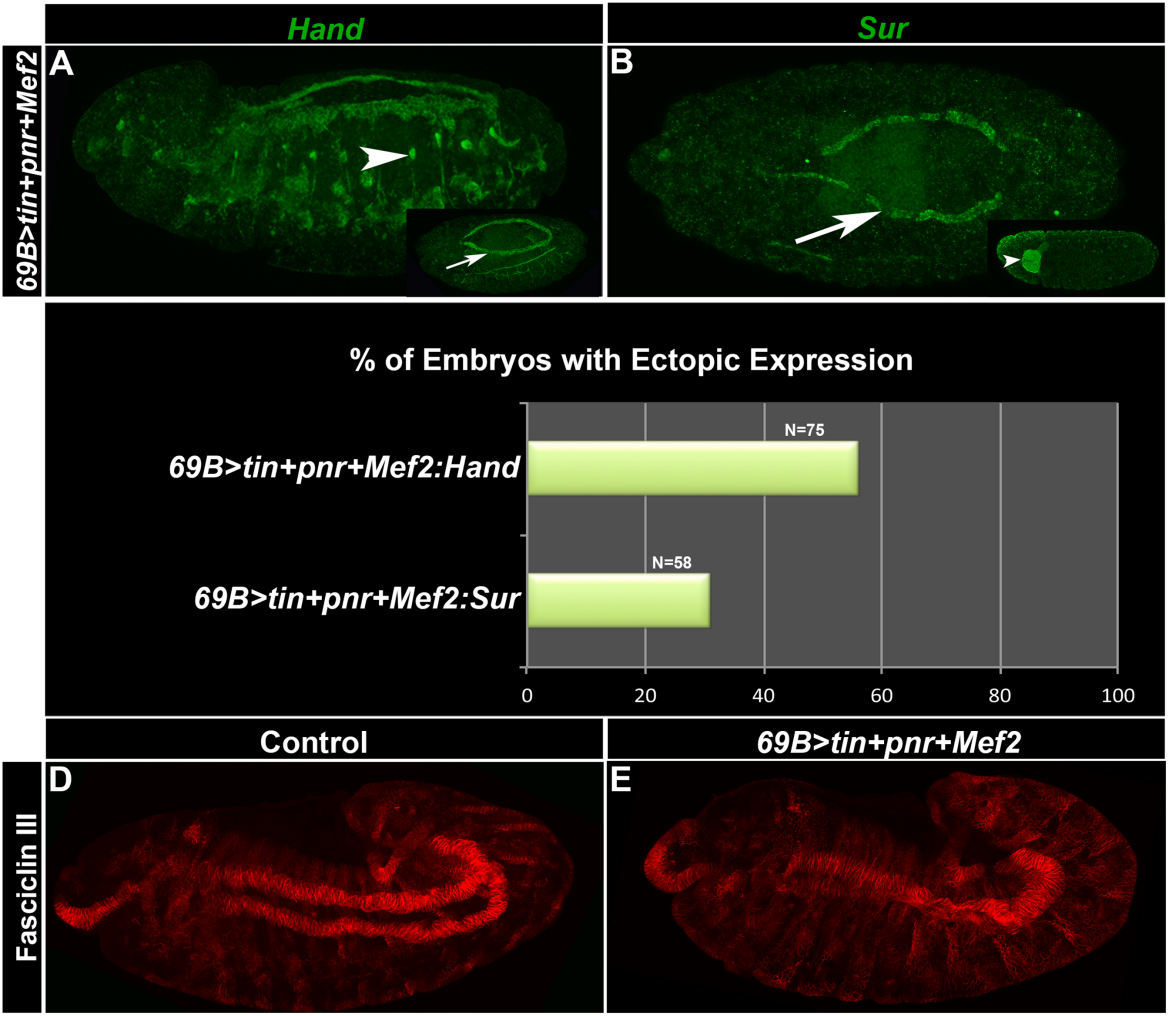
Expression of *tinman*, *pannier* and *Mef2* in the ectoderm results in an expansion of cardiac gene expression. (A,B) 69B>*tin+pnr+Mef2* embryos at stage 14, stained for *Hand* expression (A) or *Sur* expression (B). (A) ~ 50% of embryos had expanded *Hand* expression (arrowhead) with the rest demonstrating normal expression in the heart (inset, arrow). (B) ~70% of embryos had normal *Sur* expression in the heart (arrow), with a subset showing expanded *Sur* expression in the nervous system (inset, arrowhead). Bar, 100μm. (C) Quantification of effects of over-expression of cardiac transcription factors. (D,E) Antibody stain against Fasciclin III. (D) shows normal FasIII accumulation in a stage 9 control embryo. (E) shows similar levels of accumulation in a 69B>*tin+pnr+Mef2* embryo.

To determine if Tin, Pnr and MEF2 can potentiate the cardiac phenotype outside of the mesoderm, we tested whether they could activate the cardiac program in the ectoderm, using the *69B-gal4* driver line. We found that, when we used Hand as a marker of heart fate, a little over 50% of the embryos stained had ectopic stain; and when *Sur* was used as a cardiac marker, 30% of the embryos had ectopic expression in ectodermal tissues (Fig 7A and 7B). *Sur* expression was only narrowly expanded to what appeared to be malformed salivary glands, which arise from ectodermal cells. In addition, there was no expansion of FasIII accumulation in stage 12 embryos, indicating no expansion of visceral mesoderm fate in these embryos (Fig 7D and 7E). These results suggested that conversion to a cardiac fate requires either some threshold level of activation by the converting factors that may or may not be met when utilizing an ectodermal driver; or, there are additional mesodermal factors with which Tin, Pnr and MEF2 collaborate to activate the myogenic program.

## Discussion

### Activation of *Mef2* transcription by Tin and Pnr

In this paper, we characterize a *Mef2* enhancer that becomes active late in embryogenesis in all cells of the heart. We show that its activity is dependent upon a single Tin consensus binding site which is capable of binding to the Tin protein in vivo, confirming the ChIP-sequencing observations of Jin et al. indicating that this region of *Mef2* is responsive to Tin [35]. We further show that Tin can activate the enhancer in cell culture, and when the GATA transcription factor Pnr is added, there is synergistic activation of the enhancer. Similarly, ectopic expression of Tin in the ectoderm results in expansion of enhancer activity, while combined ectopic expression of Tin and Pnr dramatically expands enhancer activity as well as MEF2 expression in the ectoderm. Our results demonstrate the power of Tin and Pnr to activate *Mef2* transcription, and the importance of maintaining MEF2 at high levels in the heart through the end of embryogenesis by having duplicate enhancers with similar activity. It is interesting to note that while we did not observe a direct interaction between Pnr and the enhancer through DNA binding assays, Pnr nevertheless potentiated activation of the enhancer by Tin. This is similar to the observation of Akasaka et al [48] for activation of *Sur* by Tin and Pnr without direct binding of Pnr. However, in this instance, we did see a modest reduction in enhancer activity when we mutated the Pnr binding sites. Mechanistically, the synergistic activity probably arises from a direct interaction between Tin and Pnr that was documented by Gajewski et al [29] and can also be seen in the collaboration and direct binding of the Tin and Pnr vertebrate homologs, Nkx2.5 and GATA-4. These two transcription factors synergistically activate vertebrate cardiac enhancers independently of GATA-4 DNA binding [52–53]. The reduction of activity we saw with the mutated Pnr consensus sites suggests that Pnr may weakly interact directly with the enhancer, but the inability to demonstrate an interaction in vitro along with the above mentioned studies, leads us to hypothesize that direct interaction with Tinman is likely more important for the synergistic activation of the enhancer.

Activity of this *Mef2* enhancer in the cardiac tube is broad, including both Tin and Svp cardiac cell types. A role for Tin regulation of this enhancer might therefore seem counter-intuitive, since the enhancer is ultimately active in the Tin-negative Svp cells. A solution to this potential contradiction is that the enhancer is first active at a time when all cardiac cells are still Tin-positive, and thus Tin might function early in the activity of the enhancer to enable other cardiac factors to interact with the DNA. At later stages, when Tin is not present in all cells where the enhancer is active, the attracted factors must be able to function in activation of gene expression after Tin is no longer present. Some support for this model comes from work demonstrating that Nkx2.5 interacts with p300, a bridge to the basal transcriptional complex, which also possesses histone acetyltransferase activity. Together, p300 and Nkx2.5 synergistically activate enhancers that have been shown to be regulated by Nkx2.5 [56]. We hypothesize that recruitment of p300 and basal machinery by Tin in the early heart cells is maintained through the end of embryogenesis independently of Tin.

### *Mef2* cardiac enhancers have overlapping activities

A number of developmentally important genes, such as *Mef2*, contain multiple enhancers, often with overlapping activities. Duplicate enhancers were identified when looking for targets of Dorsal in the *Drosophila* embryo. The genes *brinker* and *sog*, for which Dorsal-dependent enhancers had already been described, were each found to have secondary enhancers in distant locations, one of which was in the intron of another gene [57]. In another example, duplicate enhancers in the *snail (sna)* gene were shown to work equally well when they were the only regulatory element controlling *sna* expression. At elevated temperatures however, there was a reduction in *sna* expression and disruptions in gastrulation when only one enhancer was supporting *sna* transcription [58], suggesting that the redundancy provides developmental insurance for the embryo during stressful environmental situations.

Given the importance of *Mef2* expression to heart muscle differentiation, it might be predicted that *Mef2* contains multiple enhancers active in the developing cardiac mesoderm. Indeed, the enhancer that we describe here is the third cardiac enhancer described for *Drosophila Mef2*, and the second to respond to Tin and Pnr. The partial temporal and spatial overlaps in enhancer activity with other *Mef2* cardiac enhancers could allow for robustness in *Mef2* transcription in the heart, essentially functioning as a shadow enhancer.

### The roles of Tin, Pnr and MEF2 in controlling cardiac fate

Vertebrate studies aimed at transforming fibroblasts into cardioblasts have relied upon cocktails of factors, in several cases of which the orthologs of Tin, Pnr and MEF2 were used [17–21]. Our data demonstrating that Tin and Pnr regulate *Mef2* expression through multiple enhancers led us to hypothesize that these factors might work together to activate the cardiac program in *Drosophila*. Prior studies in Drosophila have demonstrated that cardiac fate in the embryo can be broadened by ectopic expression of signaling factors that specify cardiac fate, including the WNT molecule Wingless, and the BMP protein Dpp [59]. These manipulations resulted in partial expansion of cardiac fate. When cardiac transcription factors were ectopically expressed, *tin* plus *pnr* expanded the expression of *Hand*, although not in all embryos [49]. Here, we demonstrate that addition of MEF2 to Tin plus Pnr significantly potentiates the activation of cardiac marker gene expression. In the case of *Sur*, a broad swath of cells is induced to express *Sur*, even though *Sur* is not thought to respond to activation by MEF2. Expression of the T-box gene H15 and the pericardial specific gene, Pericardin, were also expanded. These studies support mammalian conversion experiments indicating that MEF2 can play an important role in conversion of fibroblasts to myoblasts [17–20].

It is interesting to note that MEF2 potentiates expansion of cardiac marker gene expression in the mesoderm, where *Mef2* is already broadly expressed. This result could be explained by the need for high levels of MEF2 expression to promote cardiac fate, and the levels in the general mesoderm might not be sufficiently high to achieve conversion to cardiac fate in the absence of added MEF2. Some support for this explanation comes from the work of Gunthorpe et al [60], who showed that the differentiating heart was the most sensitive mesodermal structure to the levels of MEF2 activity, and that even slightly reduced MEF2 function resulted in a failure of normal heart differentiation.

More broadly, our data demonstrate that cardiac makers can be induced in the Drosophila system using a similar set of cardiac transcription factors that function in vertebrates. Our data further support the homology in function for cardiac factors across large evolutionary distance. In addition, our findings demonstrate that the Drosophila system can be used to unravel mechanisms of cardiac fate determination and conversion.

## Supporting Information

S1 Fig *Mef2* genetic locus, known cardiac enhancers and their regulators. (A) GBrowse image (from FlyBase.org) of the *Mef2* gene span. Known cardiac enhancers are shown as black or red boxes upstream of the A-D isoforms. (B) The cardiac cells in which each enhancer is active, known activators, and references for each enhancer.
(EPS)

S2 Fig Expression of *tinman* and *pannier* in the ectoderm results in expansion of a *Mef2* cardiac enhancer in the ectoderm. (A-D) 69B>*tin+pnr* embryo carrying the -2775/-2432 *Lmd_mut-lacZ* enhancer. A single confocal Z-slice was taken and the boxed area in (A) is enlarged in (B-D). (B) is Fasciclin III expression, (C) is β-Galactosidase expression and (A,D) are merges.
(EPS)
